# A MRI-based radiomics model for predicting the response to anlotinb combined with temozolomide in recurrent malignant glioma patients

**DOI:** 10.1007/s12672-023-00751-x

**Published:** 2023-08-23

**Authors:** Yurong Li, Weilin Xu, Yinjiao Fei, Mengxing Wu, Jinling Yuan, Lei Qiu, Yumeng Zhang, Guanhua Chen, Yu Cheng, Yuandong Cao, Shu Zhou

**Affiliations:** 1 The First School of Clinical Medicine, Nanjing Medical University, Nanjing, China; 2 Department of Radiation Oncology, Nanjing Medical University First Affiliated Hospital, Nanjing, China; 3 Department of Radiation Center, Shanghai First Maternity and Infant Hospital, Tongji University School of Medicine, Shanghai, 201204 China; 4 Nanjing Jinling Hospital, Affiliated Hospital of Medical School, Nanjing University, Nanjing, China; 5 Department of Oncology, The Second Hospital of Nanjing, Nanjing, China; 6 The Second Affiliated Hospital, Zhejiang University School of Medicine, Hangzhou, China

**Keywords:** Radiomics mode, Anlotinb, Temozolomide, Recurrent malignant glioma

## Abstract

**Objective:**

Anlotinib is a multitarget anti-angiogenic drug that combined with temozolomide (TMZ) can effectively prolongs the overall survival (OS) of recurrent malignant glioma(rMG),but some patients do not respond to anlotinib combined with TMZ. These patients were associated with a worse prognosis and lack effective identification methods. Therefore, it is necessary to differentiate patients who may have good response to anlotinb in combination with TMZ from those who are not, in order to provide personalized targeted therapies.

**Methods:**

Fifty three rMG patients (42 in training cohort and 11 in testing cohort) receiving anlotinib combined with TMZ were enrolled. A total of 3668 radiomics features were extracted from the recurrent MRI images. Radiomics features are reduced and filtered by hypothesis testing and Least Absolute Shrinkage And Selection (LASSO) regression. Eight machine learning models construct the radiomics model, and then screen out the optimal model. The performance of the model was assessed by its discrimination, calibration, and clinical usefulness with validation.

**Results:**

Fifty three patients with rMG were enrolled in our study. Thirty four patients displayed effective treatment response, showed a higher survival benefits than non-response group, the median progression-free survival(PFS) was 8.53 months versus 5.33 months (*p* = 0.06) and the median OS was 19.9 months and 7.33 months (*p* = 0.029), respectively. Three radiomics features were incorporated into the model construction as final variables after LASSO regression analysis. In testing cohort, Logistic Regression (LR) model has the best performance with an Area Under the Curve (AUC) of 0.93 compared with other models, which can effectively predict the response of rMG patients to anlotinib in combination with TMZ. The calibration curve confirmed the agreement between the observed actual and prediction probability. Within the reasonable threshold probability range (0.38–0.88), the radiomics model shows good clinical utility.

**Conclusions:**

The above-described radiomics model performed well, which can serve as a clinical tool for individualized prediction of the response to anlotinb combined with TMZ in rMG patients.

**Supplementary Information:**

The online version contains supplementary material available at 10.1007/s12672-023-00751-x.

## Introduction

According to the 2019 global Central Nervous System reported by the World Health Organization (WHO), brain tumors have the highest incidence and mortality rates in China [1]. Among primary brain tumors, gliomas are the most common intracranial malignancy in adults, accounting for approximately 50% of cases [2]. The incidence of gliomas in adults varies worldwide, based on factors such as age, sex, geographic location, and ethnicity. It ranges from 1.9 to 9.6 per 100,000 population [3, 4]. The prognosis of gliomas is influenced by several clinical factors, including sex, age and histopathological grade [5]. Maximal surgical resection and six courses of adjuvant temozolomide(TMZ) are the current standards for first-line treatment of malignant gliomas [6]. The recurrent malignant glioma (rGM) is characterized by high rates of relapse within 8 months of initial treatment [7] and poor prognosis, with a median survival ranging from 3 to 9 months [8]. Treatment options are limited, with reoperation, chemotherapy, and radiotherapy being the primary methods for managing rGM. Therefore, there is an urgent need to actively seek more effective treatment options that can improve the clinical efficacy of patients with rGM.

Despite numerous clinical trials, many second-line regimens for rGM have proven to be of limited success. Bevacizumab, a vascular endothelial growth factor(VEGF) antibody, has been shown to prolong progression-free survival(PFS) in glioblastoma [9]. However, no significant overall survival(OS) benefit was observed with bevacizumab when used alone or in combination therapy [10]. On the other hand, anlotinib, a novel tyrosine kinase inhibitor that blocks fibroblast growth factor receptor(FGFR), platelet-derived growth factor receptor(PDGFR), vascular endothelial growth factor receptor(VEGFR), and stem cell factor receptor c-kit [11], has been approved for the treatment of small cell lung cancer, non-small cell lung cancer, medullary thyroid cancer, and soft tissue sarcoma in China [12–14]. A recent study investigated the efficacy of Anlotinib, either alone or in combination with TMZ, for treating Recurrent High-Grade Gliomas (rHGGs).Among 21 patients with grade IV gliomas, the median OS and PFS were 6.2 and 4.5 months, respectively [15]. Notably, the study found that even after excluding patients with better baseline characteristics, the OS at 12 months (27.9%) and PFS at 6 months (40.2%) exceeded those reported in the TMZ re-challenge study (28.6% and 35.7%) [16]. These results suggest that Anlotinib holds promise as a potential treatment option for HGGs. Despite the potential benefits of anlotinib in combination with TMZ, some patients do not receive sufficient benefit from this therapy, and may even experience serious side effects and poor survival [12, 17, 18]. Therefore, it is crucial to identify patients who may not respond to anlotinib in combination with TMZ prior to treatment, in order to provide more personalized targeted therapy and avoid toxicity and unnecessary costs for non-responsive patients. Thus, there is a critical need to develop reliable screening methods to identify patients who are likely to benefit from this therapy, while sparing those who may not.

Radiomics, an emerging and promising field, hypothesizes that medical imaging can provide crucial information regarding tumor physiology [19–21].By converting medical images into high-dimensional, mineable, and quantitative imaging features via high-throughput extraction of data-characterization algorithms, radiomics methods provide an unprecedented opportunity to improve decision-support in oncology at low cost and noninvasively [19, 22]. Recent studies have highlighted the ability of MRI-based radiomics to predict both PFS and OS in gliomas [23]. Moreover, pretreatment MRI features have been shown to be predictive of response to Bevacizumab in patients with recurrent glioma [24]. As of now, no studies have examined the possibility of using MRI-based radiomics to predict the response to anlotinib in combination with TMZ in patients with rMG.Such investigations could provide valuable insights into personalized targeted therapy for this patient population.

Our study aimed to develop a practical model that integrates radiomics features derived from recurrent MRI scans to predict the response of rMG patients to anlotinib combined with TMZ. This approach has the potential to facilitate personalized treatment decisions based on individual patient disease characteristics.

## Materials and methods

### Participants

**Fig. 1:**
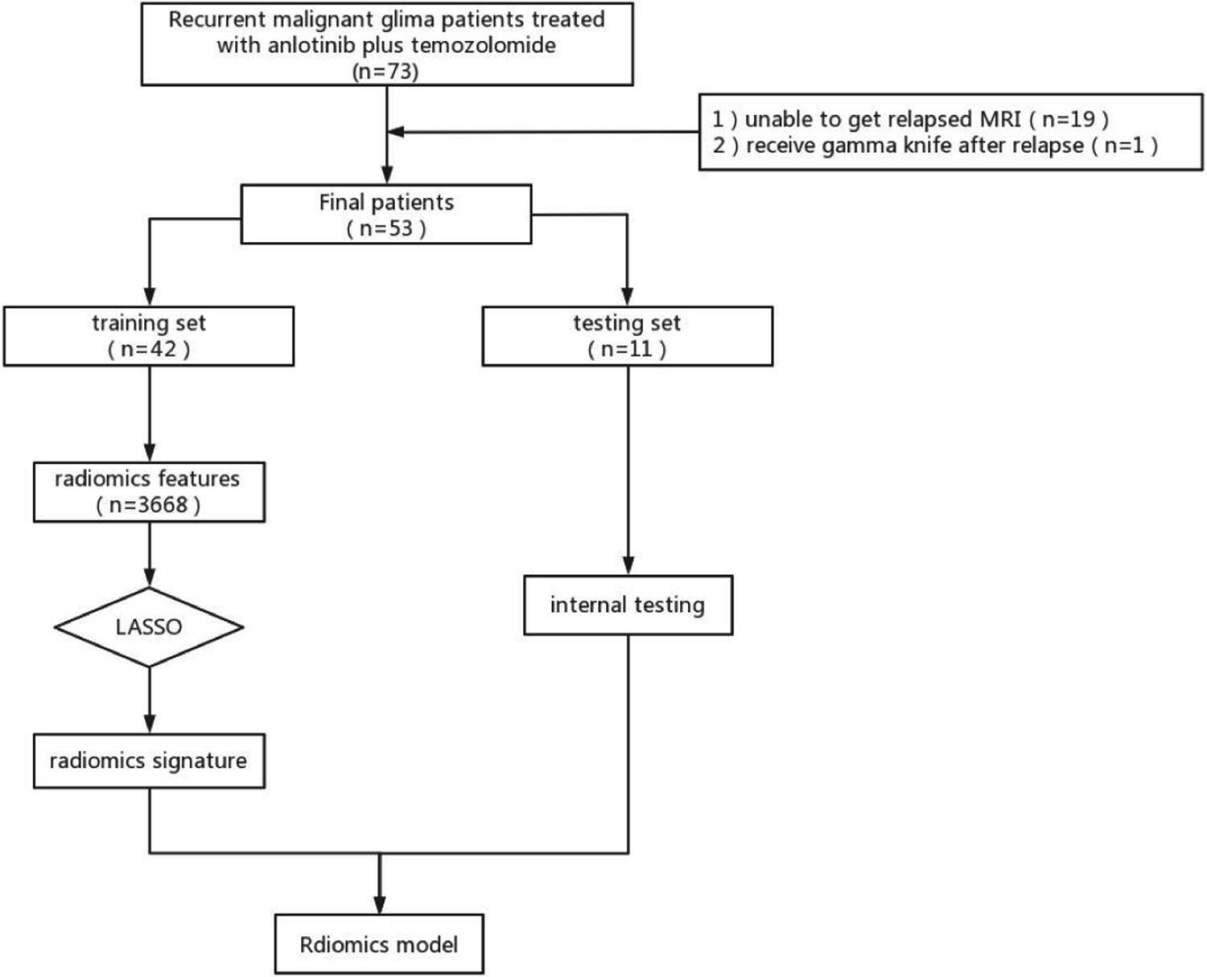
The flowchart of this study

We recruited patients who experienced treatment failure following standard STUPP regimens (Concurrent oral administration of TMZ 75 mg/m^2^ and at least 6 cycles of adjuvant chemotherapy during radiotherapy) for rGM between March 2018 and February 2022 at the Radiotherapy Center of the First Affiliated Hospital of Nanjing Medical University. Diagnosis of glioma recurrence was based on the expertise of a neurologist, radiologist, and radiotherapist. The inclusion criteria for our study were: (1) Age of 18 years or older; (2) Karnofsky Performance Status (KPS) score of  ≥ 60; (3) Pathologically confirmed diagnosis of malignant gliomas according to WHO II, III, or IV grading; (4) Presence of recurrence or residual lesions confirmed by MRI following treatment with standard STUPP regimens; (5) followed by the Response Assessment in Neuro-Oncology(RANO) criteria, which require the presence of at least one measurable lesion for accurate identification of disease progression, pseudoprogression, or radiation necrosis[25]; (6)No use of other targeted drugs during treatment; (7) No functional impairment of important organs, no other contraindications to treatment. The exclusion criteria were as follows: (1) Patients with serious underlying systemic diseases or a life expectancy of less than 3 months; (2) Incomplete clinical data that prevents effective evaluation of clinical efficacy; (3) A history of previous mental illness; and (4) Presence of other malignant tumors. The flowchart is presented in Fig. 1.

### Treatment

Patients in our study received oral administration of anlotinib once daily for 14 days every 21 days. For patients receiving intensified TMZ therapy, TMZ was administered at a dose of 100 mg/m^2^ once daily for 7 days every 14 days. Standard dosage regimens consisted of 150–200 mg/m^2^ orally for 5 days every 28 days until progression, intolerable toxicity, or death. The initial dosage of anlotinib was 12 mg. If grade 2 hemorrhage or other grade 3 or 4 adverse events occurred, the dose could be reduced to 10 or 8 mg.

### Efficacy evaluation

Patients suspected of recurrence approximately 3 months after treatment underwent dynamic observation before undergoing MRI and functional MR imaging. The assessment of recurrence criteria was mainly carried out by MDT (consisting of experts from Radiotherapy Department, Neurosurgery Department, and Radiology Department), who followed the RANO standard and comprehensively analyzed the clinical manifestations, enhancement scope, and time of occurrence of each patient. All patients underwent MRI, and during the MDT process, we evaluated whether patients needed additional functional MRI (MRS and PWI) to assist with diagnosis. On conventional MRI, the signal pattern of recurrent glioma exhibited an apparent lack of uniformity, displaying a mixture of T1/T2 signal shadows along with the presence of distinct peripheral finger edema shadows. The enhanced scan further revealed prominent abnormal enhancement in both annular and nodular forms [25]. Most recurrent gliomas appear as high perfusion on Perfusion weighted imaging (PWI) [26, 27], and the sensitivity for diagnosing glioma recurrence is notably higher when the Cho/NAA ratio in Magnetic resonance spectrum (MRS) is greater than 1.8 [28]. The primary endpoints being the objective response rate (ORR), defined as the proportion of patients with complete or partial responses and stable disease for at least 4 weeks, and the disease control rate (DCR), defined as the proportion of patients with complete or partial responses and stable disease for at least 4 weeks. Secondary endpoints included PFS and OS.

### Tumor segmentation, radiomics feature extraction and development of MRI-based radiomics model

In this study, image acquisition was performed using a 3.0 T magnetic resonance imaging scanner (Siemens MAGNETOM Vida 3.0 T MR) equipped with a cranial 8-channel orthogonal coil. All enrolled patients received routine T1-Weighted Imaging(T1WI) and T2-Fluid Attenuated Inversion Recovery(T2FLAIR) sequence scanning on transverse cephalic scan, followed by intravenous injection of contrast agent Gd⁃DTPA (0.1 mmol/kg body weight) for T1WI axial and sagittal thin layer 3D T1-weighted contrast-enhanced(T1C) scanning, coronal enhancement was reconstructed by sagittal thin layer enhancement post-processing. Conventional scan parameters were utilized with the following specifications: T1WI: TR 400 ms, TE 2.48 ms, layer thickness 5 mm, matrix 320 × 256, FOV 230 mm × 230 mm; Sagittal position thin layer 3D T1C: TR 1600 ms, TE 1.8 ms, layer thickness 1 mm, voxel size 0.7 × 0.7 × 1.0mm^3^, matrix 320 × 288, FOV 220 mm × 220 mm; T2FLAIR: TR 8000 ms, TE 97 ms, layer thickness 5 mm, layer spacing 1 mm, TI 2300 ms, Matrix 256 × 256, FOV 230 mm × 230 mm.

Two radiologists independently performed MRI feature analysis. The readers, who were blinded to the clinical-pathological data, segmented the volume of interest (VOI) using ITK-SNAP software (version 4.0; http://www.itksnap.org/pmwiki/pmwiki.php).The outermost boundaries of the tumor and edema were delineated artificially on the T1C and T2FLAIR images, respectively. If there are differences in opinions between the two radiologists, a third expert specialized in the field of gliomas was consulted. To account for variations in the MRI scanner protocols, several pre-processing steps were applied. The first step was to reorient the images and labels such that they had a consistent anatomical orientation with RAS axis codes. The next step involved resampling the images and labels using bilinear interpolation for the images and nearest neighbor interpolation for the labels to ensure a voxel spacing of 1 × 1x1 mm^3^. Then, the intensity values of the images were rescaled to have a range of [0,1] using a linear transformation, with optional clipping. Finally, removal of the background region of the images and labels was performed based on the foreground mask computed from the source image.

All radiomics signatures were extracted using Pyradiomic's (version 3.0.1; http://pyradiomics.readthedocs.io) in-house feature analysis procedure (Supplementary document). For each MRI sequence, we extracted 7 feature groups, shape (28 features), first-order statistical features (720 features), NGTDM (200 features), GLCM (880 features), GLDM (560 features), GLRLM (640 features) and GLSZM (640 features).

**Fig. 2:**
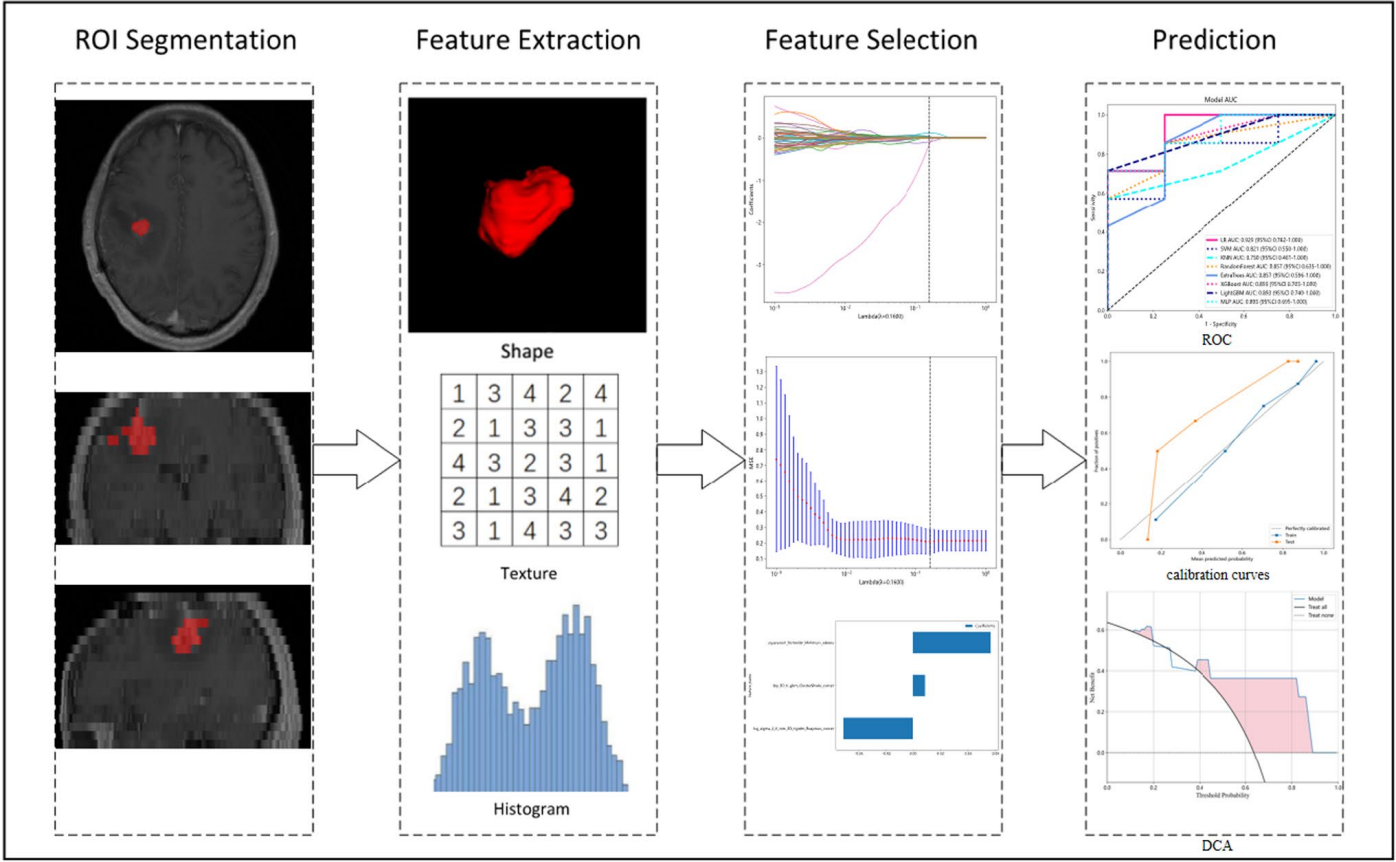
Workflow of radiomic analysis of this study

Firstly, characteristics with stability and repeatability were selected using Spearman's rank correlation coefficient test. Next, all features were standardized using the Z-score method, and hypothesis testing was employed to screen out features with significant differences between the two groups to ensure the validity of all features. Feature dimensionality reduction was achieved using Least Absolute Shrinkage And Selection Operator (LASSO) Regression, which can effectively reduce overfitting and improve prediction accuracy. The regularization parameter (λ) in feature selection was adjusted by tenfold cross-validation of the minimum criteria. Radiomics signatures were developed using selected features with nonzero coefficients. To build a predictive model of radiomics in a supervised learning manner, Support Vector Logistic Regression (LR), Machine (SVM), K-Nearest Neighbor (KNN), Extremely Randomized Trees (Extra Trees), Random Forest (RF), eXtreme Gradient Boosting (XGBoost), Light Gradient Boosting Machine (LightGBM), and Multilayer Perceptron (MLP) classifiers were utilized in the training cohort. fivefold cross verification was used to select the best performance model. Firstly, multiple model choices are verified on the training set and testing set, and the model with minimum average error is selected. After selecting the appropriate model, the training set can be combined with the testing set, and the model can be trained again on it to get the final model, and then the test set can be used to test its generalization ability. The radiomic score (Rad-score) was calculated by a linear combination of selected features weighted by their respective coefficients. Model evaluation was performed using Area Under the Curve (AUC) values, accuracy, specificity, and sensitivity. Finally, the application value of the final model was analyzed through Decision Curve Analysis (DCA).Fig. 2 provides an illustration of the image analysis workflow in our study.

### Statistical methods

Statistical analyses in this study were performed using IBM SPSS Statistics software(version 25.0, https://www.ibm.com) and R statistical software(version 3.3.3, https://www.r-project.org). Survival analyses were conducted using the Kaplan–Meier method with a 95% confidence interval and compared using the Breslow test. The features were analyzed using Mann–Whitney U-test, t-test, or χ2 test, with statistical significance set at *p* < 0.05.For features with high repeatability, Spearman's rank correlation coefficient was also applied to calculate the correlation between features, and features with a correlation coefficient greater than 0.9 between any two features were retained. To maximize the ability to describe features, a greedy recursive deletion strategy was utilized for filtering features, which involved deleting the most redundant features in the current set each time. LASSO regression modeling was performed using the Python scikit-learn package.

## Results

### Patient characteristics

Between March 2018 and February 2022, a total of 73 patients with rMG from the Radiotherapy Center at the First Affiliated Hospital of Nanjing Medical University were initially included in the study. After excluding 19 patients who were unable to undergo MRI and 1 patient who underwent gamma knife therapy after relapse, 53 patients (33 male and 20 female) were ultimately included in the analysis. The median age of the patients was 64 years (range: 25–77), and 46 cases had KPS scores greater than or equal to 90. Of the 53 patients, 5 had grade II gliomas (2 oligodendroglioma, 3 astrocytomas), 9 had grade III gliomas(4 anaplastic astrocytomas, 5 anaplastic oligodendrogliomas), and 39 had grade IV gliomas. Most patients had negative prognostic factors, such as isocitrate dehydrogenase (IDH) wild type(60.4%). Ten patients had macroscopic tumor residue after surgery, and 6 patients had multifocal or disseminated tumors. For the analysis, the 53 rMG patients were divided into response and non-response groups(34vs19), and baseline characteristics such as age, sex, KPS, Body Mass Index (BMI), histology grade, multifocality, and tumor and edema volume were evaluated. Results of the between-group comparisons (*p* > 0.05) indicated that there were no significant differences between the two groups, that we will conduct further analysis after increasing the sample size in the future. Table 1 describes the clinical data of the study.

**Table 1:** Baseline clinical characteristics of patients

Features	All	Non-response	Response	P value
Age	53.51 ± 11.28	55.74 ± 10.06	52.26 ± 11.86	0.287
Original_BMI	23.86 ± 2.94	24.13 ± 3.21	23.71 ± 2.81	0.62
Recurrent_BMI	24.32 ± 2.65	24.61 ± 3.04	24.16 ± 2.44	0.554
Gender				0.115
0	20 (37.74)	4 (21.05)	16 (47.06)	
1	33 (62.26)	15 (78.95)	18 (52.94)	
Underlying disease				0.938
0	38 (71.70)	13 (68.42)	25 (73.53)	
1	15 (28.30)	6 (31.58)	9 (26.47)	
KPS				0.676
70	3 (5.66)	2 (10.53)	1 (2.94)	
80	4 (7.55)	1 (5.26)	3 (8.82)	
90	27 (50.94)	9 (47.37)	18 (52.94)	
100	19 (35.85)	7 (36.84)	12 (35.29)	
WHO				0.202
II	5 (9.43)	null	5 (14.71)	
III	9 (16.98)	4 (21.05)	5 (14.71)	
IV	39 (73.58)	15 (78.95)	24 (70.59)	
IDH status	0.26 ± 0.40	0.30 ± 0.44	0.23 ± 0.38	0.518
Residual tumor				0.95
0	43 (81.13)	16 (84.21)	27 (79.41)	
1	10 (18.87)	3 (15.79)	7 (20.59)	
Multifocal lesion				0.752
0	47 (88.68)	16 (84.21)	31 (91.18)	
1	6 (11.32)	3 (15.79)	3 (8.82)	
Confirmed cancer volume	36361.33 ± 26410.80	30514.11 ± 21562.71	39628.90 ± 28545.4	0.232
Confirmed edema volume	53731.43 ± 39207.96	64956.92 ± 40652.37	47458.36 ± 37521.10	0.12
Recurrent cancer volume	22948.11 ± 26690.09	24363.16 ± 27649.95	22157.35 ± 26527.35	0.776
Recurrent edema volume	66452.64 ± 58271.21	81498.42 ± 61376.56	58044.71 ± 55611.09	0.162

*BMI* Body Mass Index, *KPS* Karnofsky Performance Status, *WHO* World Health Organization, *IDH* isocitrate dehydrogenase

### Efficacy

**Fig. 3:**
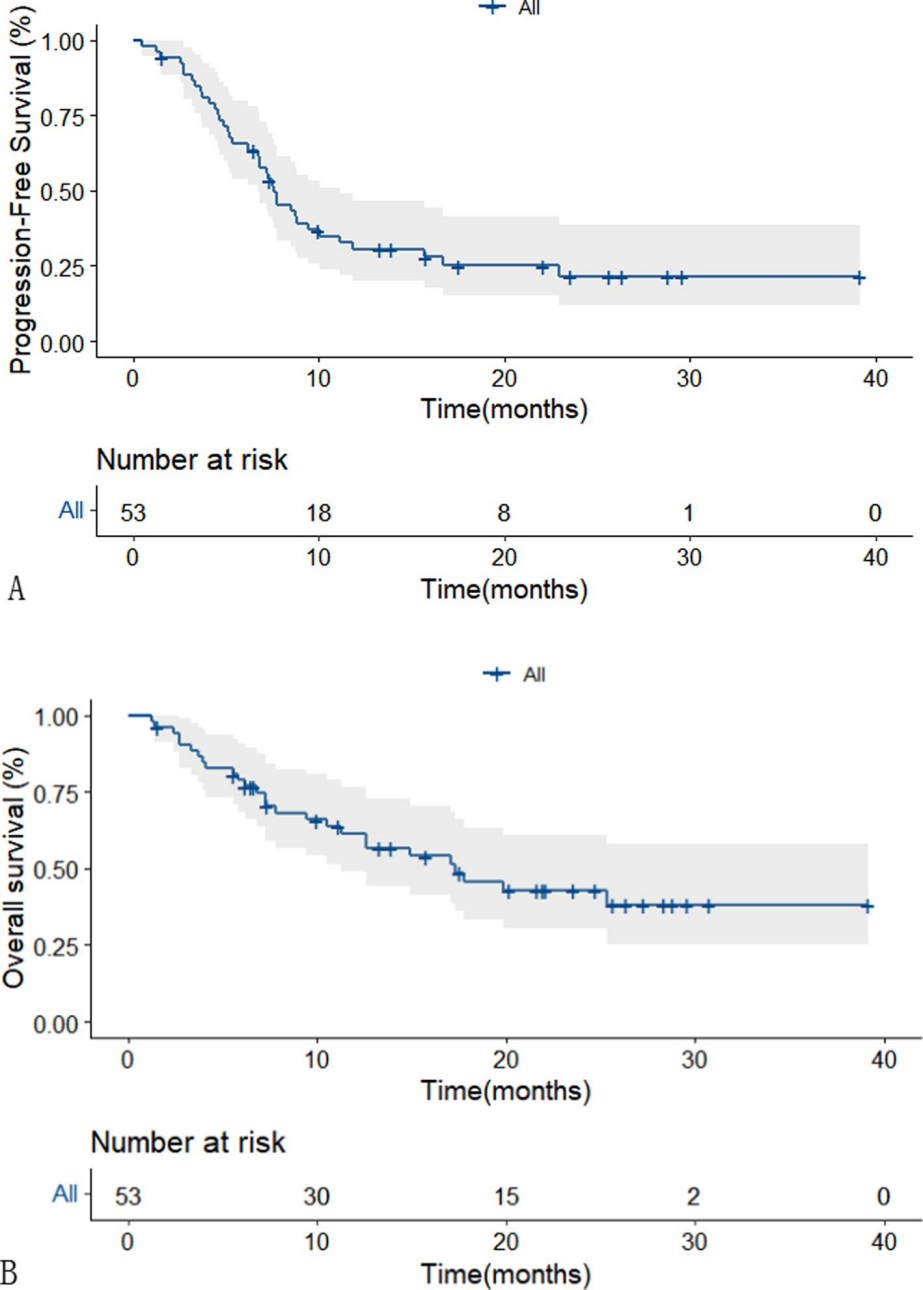
Kaplan–Meier plot of PFS in all patients.(A) Kaplan–Meier plot of OS in all patients.(B)

**Fig. 4:**
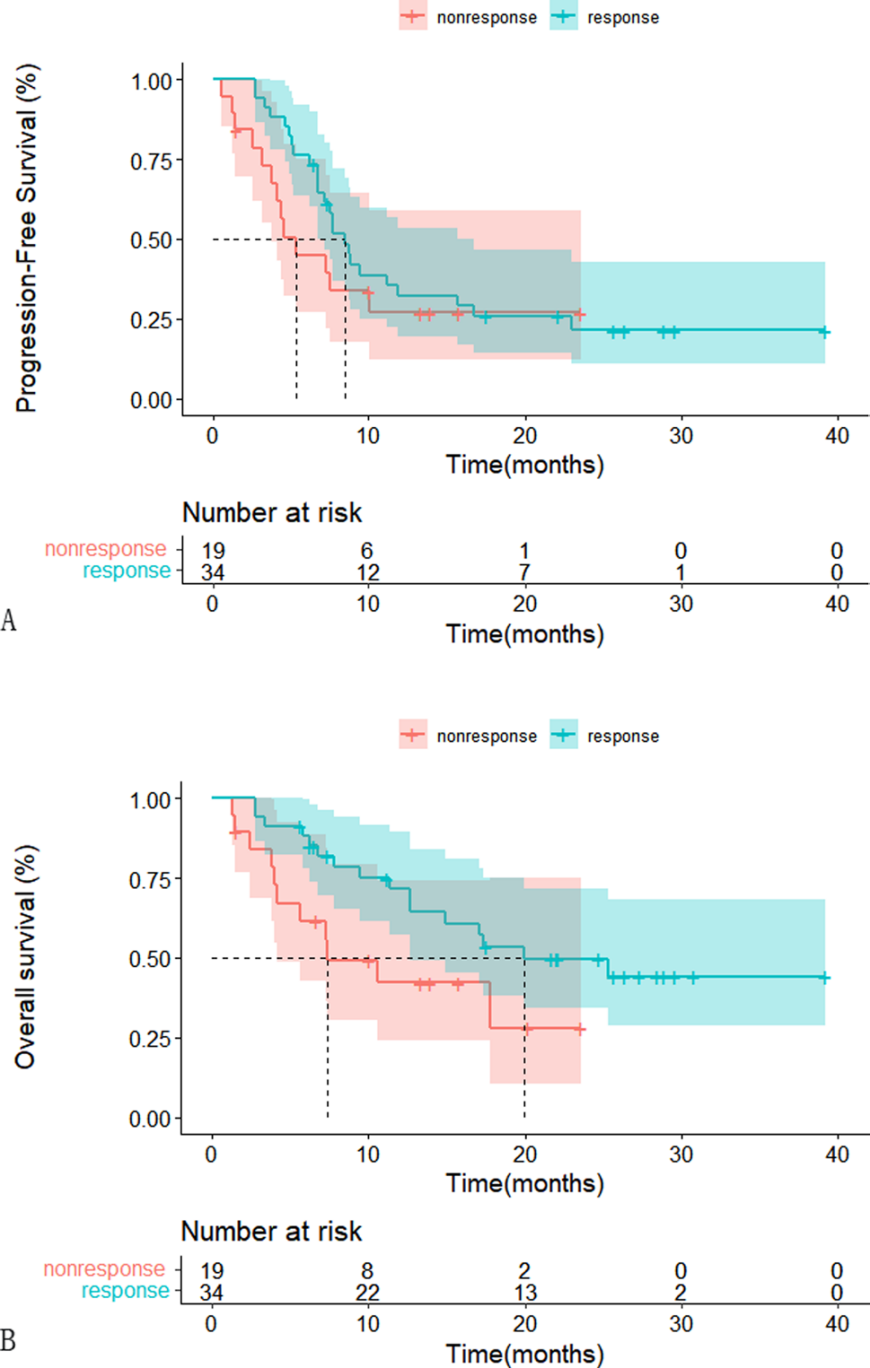
Kaplan–Meier plot of PFS of response or non-response group. (**A**) Kaplan–Meier plot of OS of response or non-response group. (**B**)

The deadline for follow-up in this study was November 1, 2022, with a median follow-up of 22.1 months (95% CI 19.27–24.93). Of the 53 patients analyzed, 11 developed disease progression (3 grade II, 1 grade III, and 7 grade IV) and 27 died(5 grade III and 22 grade IV). Partial remission was achieved in 34 patients (64.15%), and 8 patients (15.09%) had stable disease. The overall response rate(ORR) and disease control rate(DCR) were 64.15% and 79.24%,respectively.The median PFS for all patients was 7.57 months(95% CI 6.1–9.1) (Fig. 3A), with PFS rates at 6 and 12 months of 65.5% and 30.6%, respectively. The median OS was 17.3 months(95% CI 9.47–25.13) with a 61.4% survival rate at 12 months (Fig. 3B).The median duration of response was 6.8 months(95% CI 4.38–9.22).Comparison of the non-response group versus response group showed that the median PFS was 5.33 months(95% CI 3.49–7.17) and 8.53 months(95% CI 7.14–9.92; *p* = 0.06)(Fig. 4A), while the median OS was 7.33 months(95% CI 1.56–13.1) and 19.9 months(95% CI 8.27–31.53, *p* = 0.029), respectively. These results suggest that patients who are likely to have a good response to this therapeutic regimen may experience clear survival benefits (Fig. 4B).

### Acquisition of radiomic features and construction of models

**Fig. 5:**
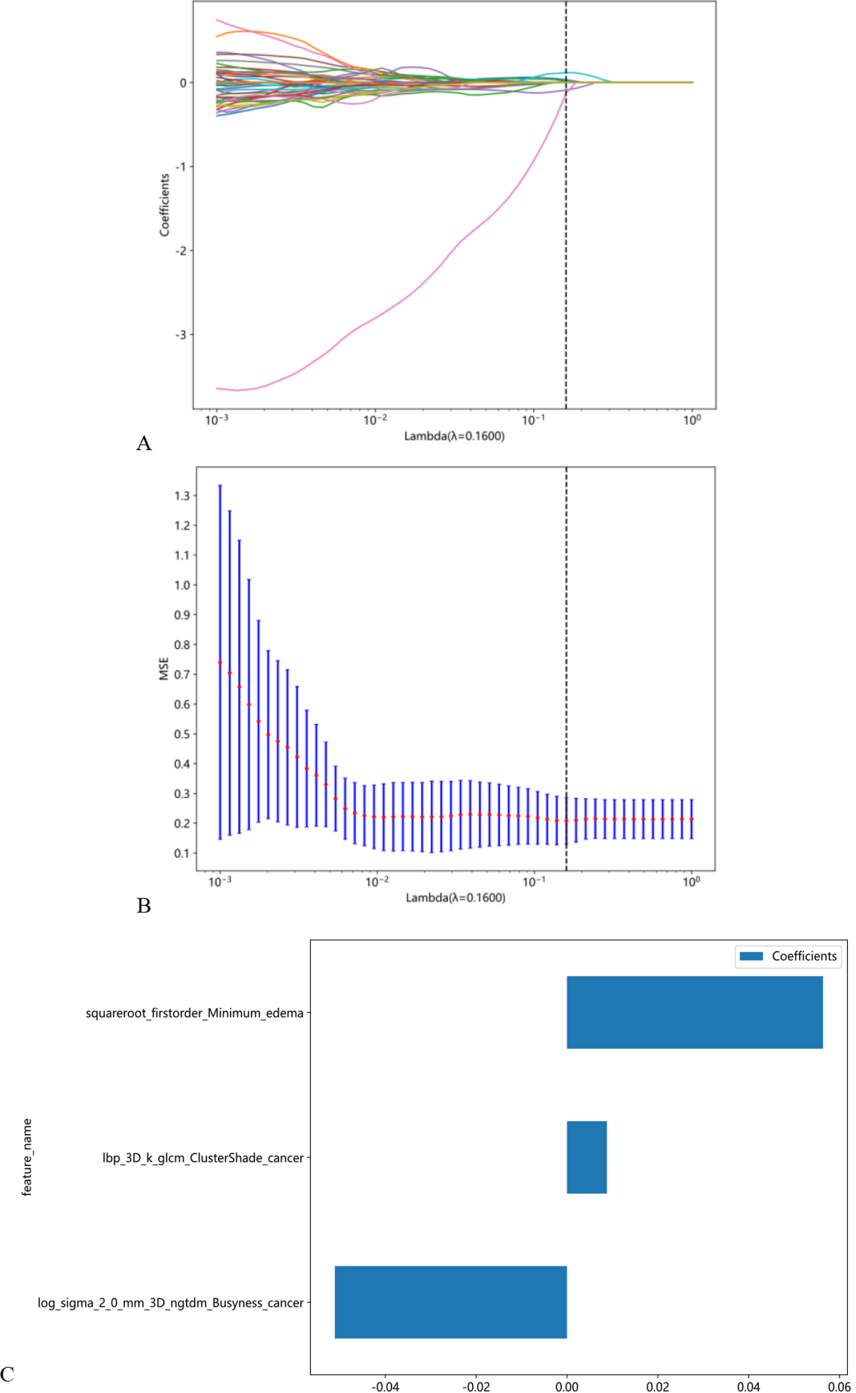
Radiomic feature selection and establishment of Rad score based on LASSO algorithm.10 fold cross-validation coefficients and MSE (**A**, **B**). Rad score histogram based on the selected features. (**C**)

The MRI images of rMG patients were analyzed using radiomics, and for each MRI sequence, 7 feature groups were extracted, including shape (28 features), first-order statistical features (720 features), NGTDM (200 features), GLCM (8880 features), GLDM (560 features), GLRLM (640 features), and GLSZM (640 features). The LASSO regression model was used to establish the Rad-score, with three non-zero coefficients selected. Altogether, a total of 3668 radiomic features were extracted for analysis. To reduce redundancy and improve model accuracy, a filtering process was used to eliminate low-variance features, resulting in 135 remaining radiomic features. Then, features with a correlation coefficient greater than 0.9 between any two features were identified and only 49 features from each highly correlated pair were retained, resulting in a final set of 3 radiomic features for model construction (Fig. 5C). The 10 folds verified coefficients and Mean Standard Error (MSE) are show in Fig. 5A and B. These characteristics are included in the calculation of Rad-score as follows:

Rad-score = 

0.708030547579847 + 0.008779*lbp_3D_k_glcm_ClusterShade_cancer-0.051104*log_sigma_2_0_mm_3D_ngtdm_Busyness_cancer + 0.056356*squareroot_firstorder_Minimum_edema.

### Predictive validity of the model

**Fig. 6:**
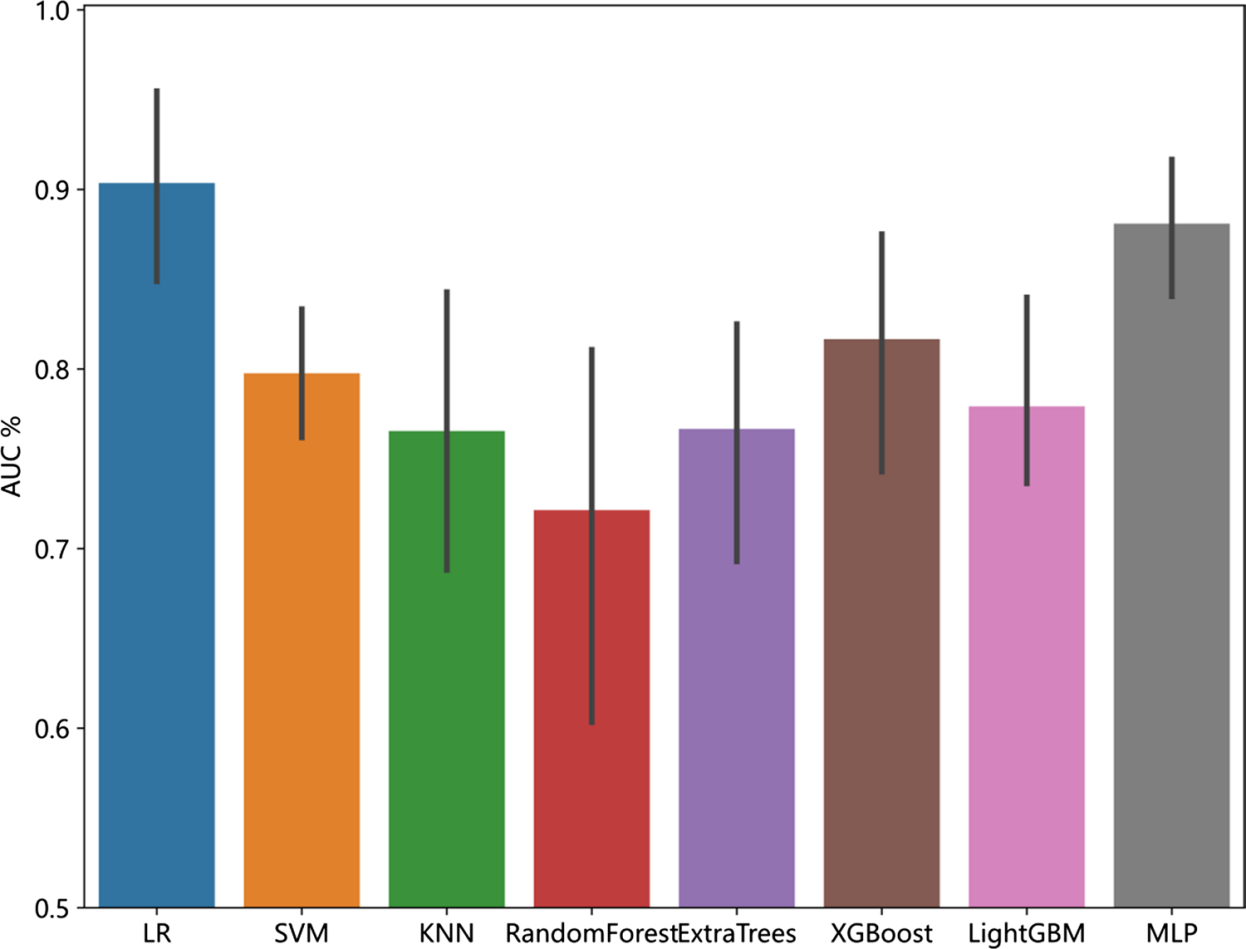
The result of fivefold cross verification

**Fig. 7:**
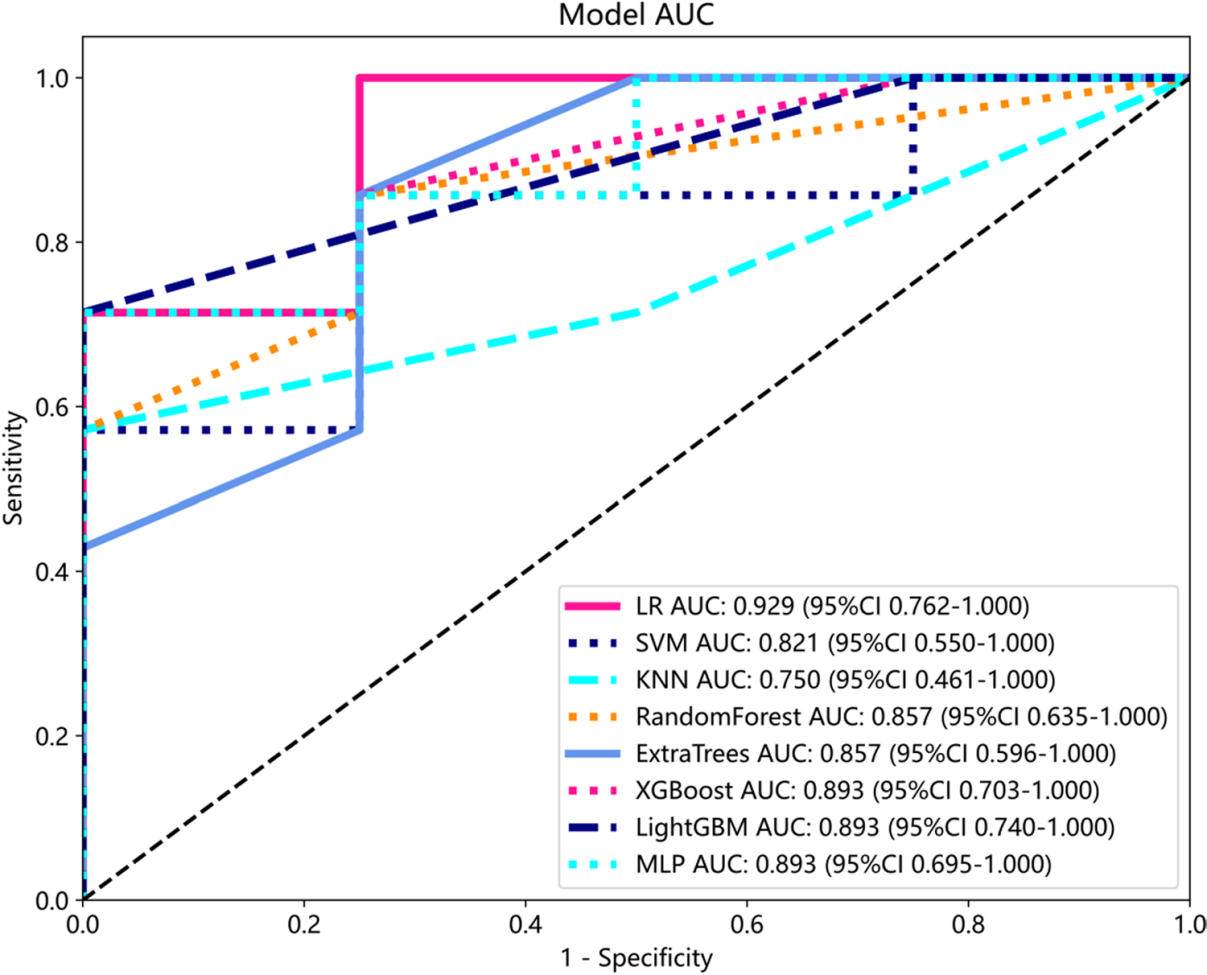
Comparison of radiometric feature model predictions for the testing cohorts

Compared to other models, LR model has the best performance. Here, we employ fivefold cross verification to obtain the final Rad signature (Fig. 6). Figure 7 showed ROC analysis of radiomic signatures by different models in the testing cohort. Machine learning methods, including LR, SVM, KNN, Random Forest, Extra Trees, MLP, XGBoost, and LightGBM, were used to construct predictive models using the radiomic features. For the training cohort, the area under the curve (AUC) values for the LR, SVM, KNN, Random Forest, Extra Trees, XGBoost, LightGBM, and MLP models were 0.906, 0.891, 0.896, 1.000, 1.000, 0.981, 0.827 and 0.904 respectively. For the testing cohort, the AUC values were0.929, 0.821, 0.750, 0.857, 0.857, 0.893, 0.893 and 0.893, respectively (Table 2). Among these models, the LR model performed the best. To obtain the final Rad signature, fivefold cross-validation was employed (Fig. 6), and Receiver Operating Characteristic (ROC) analysis of radiomic signatures by different models in the testing cohort is shown in Fig. 7.

**Table 2:** Performance of the eight classifiers in different data cohort

Model	ACC	AUC	95% CI	Sensitivity	Specificity	PPV	NPV	Threshold	Task
LR	0.857	0.906	0.8176–0.9947	0.926	0.733	0.862	0.846	0.516	Train
	0.909	0.929	0.7619–1.0000	1.000	0.750	0.875	1.000	0.192	Test
SVM	0.881	0.891	0.7694–1.0000	0.889	0.867	0.923	0.812	0.681	Train
	0.818	0.821	0.5503–1.0000	0.857	0.750	0.857	0.750	0.406	Test
KNN	0.810	0.896	0.8057–0.9869	0.741	0.933	0.952	0.667	0.800	Train
	0.727	0.750	0.4614–1.0000	0.571	1.000	1.000	0.571	1.000	Test
Random forest	1.000	1.000		1.000	1.000	1.000	1.000	1.000	Train
	0.818	0.857	0.6349–1.0000	0.857	0.750	0.857	0.750	0.818	Test
ExtraTrees	1.000	1.000		1.000	1.000	1.000	1.000	1.000	Train
	0.818	0.857	0.5960–1.0000	0.857	0.750	0.857	0.750	0.818	Test
XGBoost	0.952	0.981	0.9492–1.0000	0.926	1.000	1.000	0.882	0.601	Train
	0.818	0.893	0.7033–1.0000	0.714	1.000	1.000	0.667	0.497	Test
LightGBM	0.738	0.827	0.7045–0.9498	0.704	0.800	0.864	0.600	0.637	Train
	0.818	0.893	0.7403–1.0000	0.714	1.000	1.000	0.667	0.713	Test
MLP	0.833	0.904	0.8142–0.9932	0.815	0.867	0.917	0.722	0.681	Train
	0.818	0.893	0.6949–1.0000	0.714	1.000	1.000	0.667	0.494	Test

*LR* logistic regression, *SVM* support vector machine, *KNN* K nearest neighbor, *Extra Trees* extremely randomized trees, *XGBoost* eXtreme Gradient Boosting, *LightGBM* light gradient boosting machine, *MLP* multilayer perceptron, *ACC* accuracy, *AUC* area under curve, *PPV* positive prediction value, *NPV* negative prediction value

**Fig. 8:**
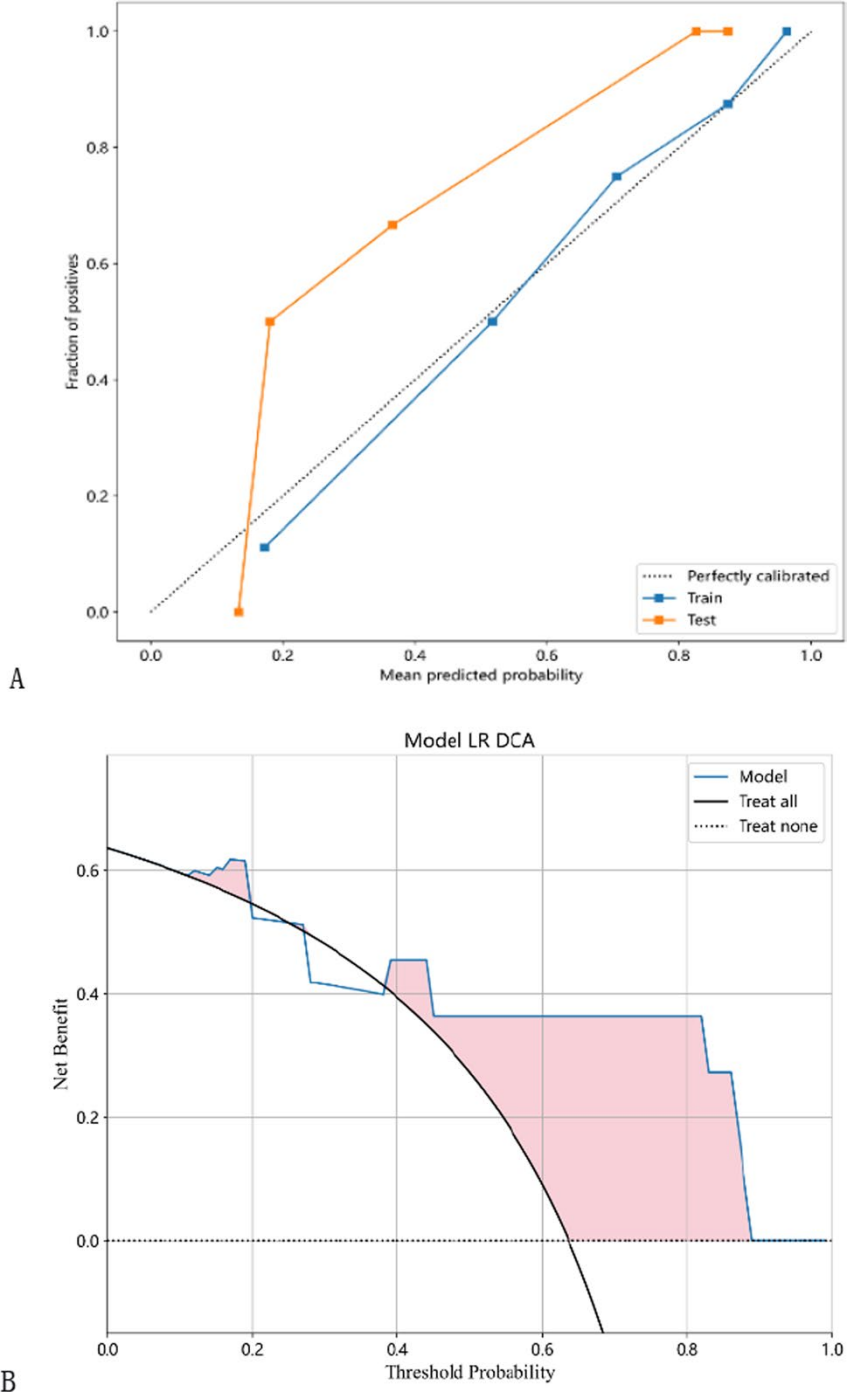
The calibration curves in the training and testing cohorts show that the radiomic model fits well in both the training and testing cohorts (**A**). Decision curves of the radiomic model in the testing cohort (**B**)

To evaluate the accuracy of the LR model, a calibration curve was plotted to compare the predicted and observed probabilities of the radiomic features in the training and validation sets (Fig. 8A). The good consistency between the observed and predicted probabilities indicates the reliability of the model. Furthermore, decision curve analysis was performed to assess the clinical utility of the predictive model. In Fig. 8B, the blue curve represents two extreme scenarios where the net benefit of treating patients is higher than the gray horizontal line (in which no patients received treatment) and the black inverted curve (in which all patients received treatment) within the threshold range of 0.38 to 0.88. The results suggest that the model has potential clinical utility in identifying patients who are likely to benefit from treatment.

## Discussion

To date, limited studies have investigated the treatment of rMG with anlotinib. Previous studies have primarily focused on evaluating the efficacy of anlotinib in combination with TMZ in rMG patients, without utilizing models to screen out patients who may have a poor response [15, 29]. In our study, we developed a practical, non-invasive MRI-based radiomics model for predicting individualized responses to the combination therapy of anlotinib and TMZ in rMG patients. Our approach enables personalized targeted therapies and reduces the risk of serious side effects and high costs for patients who may have poor response rates. Our results demonstrate that our model effectively predicts the response of rMG patients to anlotinib combined with TMZ, with a predictive efficacy of 0.93 and prediction accuracy of 0.91. This model serves as an illustrative example of precision medicine and can significantly impact treatment strategies.

Patients with rMG have a poor prognosis, particularly those who have received extensive treatment. The median PFS of rMG patients receiving doses-intensive TMZ was reported to be only 3.5 months [16].Currently, chemotherapy remains the primary treatment for rMG, while anti-angiogenesis therapy is the only recognized targeted therapy for high-grade gliomas(HGGs) [30]. Anlotinib, a multi-kinase inhibitor targeting both tumor cell proliferation and angiogenesis, has shown promise in recent studies [11]. In our retrospective study, the median PFS and OS of rMG patients treated with anlotinib in combination with TMZ were 7.57 months and 17.3 months, respectively. Among the 39 patients with grade IV disease, the median PFS and OS were 7.2 months and 12.63 months, respectively. Notably, the 6-month PFS and 12-month OS rates were 63.2% and 51.3%, respectively, which exceeded those reported in the TMZ re-challenge study (35.7% and 28.6%) [16]. Another retrospective analysis of anlotinib alone or in combination with TMZ for relapsed HGGs showed a median PFS of 4.5 months and a median OS of 7.7 months. Among the 21 patients with grade IV disease, the median PFS was 4.5 months, median OS was 6.2 months, 6-month PFS was 40.2%, and 12-month OS was 27.9% [15]. Our study demonstrated significantly better outcomes compared to the previous study, which may be attributed to our superior participant baseline characteristics, including 46 patients with a KPS of  ≥ 90, fewer multifocal lesions (11.3% vs 71%), and the inclusion of five patients with grade II glioma (9.43%). In contrast, the prior study had a higher percentage of grade IV patients (86.7% vs. 73.58%) and fewer IDH mutations (13.04% vs 20.75%). Moreover, our study had a larger sample size and utilized a uniform treatment regimen. We also found that PFS (8.53 months vs 5.33 months; *p* = 0.06) and OS (19.9 months vs 7.33 months; *p* = 0.029) in the response group were significantly longer than those of the non-response group (*p* < 0.05). Our results suggest that patients who respond to this treatment regimen have a clear survival benefit.

Radiomics has emerged as a valuable tool throughout the entire tumor treatment process. It has been widely used in anti-angiogenic drugs and brain tumor imaging; however, most studies have primarily focused on rMG and gastrointestinal tumors using bevacizumab as the primary targeted drug. Several studies have predicted the efficacy of bevacizumab in recurrent glioma using MRI-based radiomics and achieved good predictive efficacy for PFS and OS [24, 25]. Despite being limited in number, radiomics studies investigating the efficacy of small molecule anti-angiogenic drugs have shown potential for predicting drug response. For example, renal cancer radiomics based on positron emission tomography (PET), computed tomography(CT), and MRI has shown promising results in predicting early remission and survival with sunitinib, although most of these studies have been conducted on small samples [31–33]. Another study explored CT-based radiomics to predict the efficacy of anlotinib in advanced non-small cell lung cancer, also achieving promising results [34].

We developed and validated a radiomics model based on features extracted from two MRI sequences (T1C and T2FLAIR). The model achieved high classification accuracy in both the training and validation cohorts. However, our study had a relatively small sample size, which could potentially lead to overfitting of the model. To reduce this risk, we calculated the correlation between features using Spearman's rank correlation coefficient and applied a greedy recursive deletion strategy for feature filtering. We also utilized LASSO regression to screen out features. LASSO selects sample data based on punishment method, compresses coefficients, compresses originally small coefficients to 0, and thus treats variables corresponding to these coefficients as non-significant variables and directly dismisses them, while variables not with 0 coefficients are retained as final variables to achieve the purpose of feature screening. A total of three optimal features were selected for development. Different machine learning classifiers can produce varying results with the same radiomic feature set. In our study, we utilized eight different classifiers(LR, SVM, KNN, Random Forest, Extra Trees, MLP, XGBoost, and LightGBM) to model classification. These classifiers have different internal algorithms that classify samples from various perspectives. We selected the most efficient LR model for our analysis. The LR classifier demonstrated high performance, with AUC values of 0.906 and 0.929 in the training and validation cohorts, respectively. We also observed the sensitivity (0.926 and 1) and the specificity(0.733 and 0.75) between the two cohorts. fivefold cross verification results show that LR model has the best performance. The AUC is a common index used to evaluate discrimination, while calibration reflects the level of agreement between actual observed outcomes and the model's predicted outcomes.However, it is important to note that the AUC only measures the predictive accuracy of the signatures and does not determine whether the model is actually useful or worth implementing in clinical practice. DCA is a statistical method that considers the consequences of making a decision based on a given model.To complement the findings of the AUC, we performed a DCA to evaluate the clinical value of our model. The AUC and calibration curves demonstrated high prediction accuracy of our proposed radiomics model. Additionally, the DCA curve showed that our model could be used to improve treatment decision-making processes over relatively large thresholds. These results suggest that the LR model has potential and value for non-invasive prediction of rMG patients' response to androtinib combined with TMZ before treatment. Therefore, it can serve as an auxiliary tool in clinical work.

Although our study has several strengths, it also has some limitations. Firstly, Our study includes recurrent glioma patients who were initially diagnosed with grade II, III or IV in order to ensure the stability of the model and the reliability of the results. In future research, expanding the sample size and performing stratified analysis will be the focus. Therefore, it is necessary to conduct further investigations with larger sample sizes to verify our findings. Secondly, Compared to other studies, our research included more patients with a good baseline physical condition [29], fewer patients with multifocal lesions [15], and 5 patients with grade II gliomas [35], which may have affected the PFS and OS benefits of the patients. This suggest that the model we developed may be better suited for the mentioned patients, which may limit the generalizability of the radiomics model to other patient populations or clinical settings. In the future, we plan to construct a more generalizable and practical model with larger sample sizes to verify our findings. Thirdly, no external validation was not performed, which may limit the generalizability of our results. Fourthly, we manually delineated the VOI, which has a strong dependence on the operator and poor reproducibility. Currently, there are studies showing that automatic or semi-automatic methods are effective and feasible ways to reduce operator interaction in the segmentation process and to improve the reproducibility of radiomics research [36]. Lastly, we did not include clinical factors in the radiomics model because no clinically significant factors were analyzed. In future research, we will consider including meaningful clinical factors to improve the performance of the radiomics model.

## Conclusion

We developed and validated a radiomics model to differentiate rMG patients who are likely to have a good response to anlotinib in combination with TMZ from those who are not. The radiomics model demonstrated high performance and could serve as a valuable clinical tool for personalized targeted therapies. This approach would potentially spare patients who may have a poor response from experiencing serious side effects and high costs associated with ineffective treatments.

### Supplementary Information

Additional file1 (YAML 4 KB)

## Data Availability

The original contributions presented in the study are included in the article material. Further inquiries can be directed to the corresponding author.
